# “Ozempic Face”: An Emerging Drug-Related Aesthetic Concern and Its Treatment with Endotissutal Bipolar Radiofrequency (RF)—Our Experience

**DOI:** 10.3390/jcm14155269

**Published:** 2025-07-25

**Authors:** Luciano Catalfamo, Francesco Saverio De Ponte, Danilo De Rinaldis

**Affiliations:** Department of Biomedical, Dental and Morphological and Functional Imaging (BIOMORF), University of Messina, Via Consolare Valeria 1, 98124 Messina, Italy; lcatalfamo@unime.it (L.C.); lc.lab@hotmail.it (F.S.D.P.)

**Keywords:** Ozempic face, radiofrequency, diabetes mellitus, GLP-1 agonist receptor, antidiabetic, facial aging, non-surgical lifting

## Abstract

**Background/Objectives:** “Ozempic face” is an aesthetic side effect associated with the use of the antidiabetic agent Ozempic (semaglutide), characterized by a prematurely aged and fatigued facial appearance due to rapid weight loss. Currently, treatment options for this condition are limited. In this study, we present our clinical experience with the *BodyTite* device, provided by *InMode Italy S.r.l.* (Rome, Italy). **Materials and Methods:** We report a case series involving 24 patients (19 women and 5 men, aged 27–65 years), treated with subdermal bipolar radiofrequency (Endotissutal Bipolar Radiofrequency) between 2023 and 2024. All patients underwent a minimum follow-up of 12 months. At the end of the follow-up period, patients rated their satisfaction on a from 0 to 10 scale, and an independent expert assessed the stability of clinical outcomes. **Results:** The majority of patients reported high satisfaction levels (≥8), which correlated with the independent expert’s evaluation of treatment efficacy and result stability. The only observed adverse event was transient cutaneous erythema. **Conclusions:** “Ozempic face” is an increasingly common side effect associated with newer classes of antidiabetic medications. Although these drugs offer significant metabolic benefits, the accompanying facial volume loss and aging are often poorly tolerated by patients. Our findings suggest that subdermal bipolar radiofrequency represents a safe, low-risk, and cost-effective therapeutic option for the aesthetic management of Ozempic face.

## 1. Introduction

The term *“Ozempic face”* refers to a side effect associated with the use of the antidiabetic medication Ozempic (semaglutide). First described in 2023 by prominent American dermatologist Dr. Paul Jarrod Frank, the condition is characterized by a marked reduction in facial volume and soft tissue definition, primarily resulting from the rapid weight loss induced by semaglutide therapy.

More recently, however, the term has been adopted more broadly to describe the facial changes commonly observed following any form of significant and rapid weight loss, irrespective of the underlying cause. This includes weight loss resulting from other pharmacological agents or from non-pharmacological interventions, such as caloric restriction or lifestyle modification without medical supervision.

Ozempic is the commercial name for semaglutide, a recently introduced antidiabetic and hypoglycemic agent that functions as a glucagon-like-peptide-1 receptor agonist (GLP-1 RA).

Semaglutide exerts a dual pharmacological effect:

- Regulation of glucose metabolism;
- Promotion of weight loss.

The weight loss induced by semaglutide is typically rapid and affects the entire body, including the face. Although generally regarded as a desirable outcome by most patients, the facial soft tissues often fail to adapt proportionally to the sudden reduction in volume. Specifically, the skin’s intrinsic remodeling capacity is unable to keep pace with the accelerated depletion of subcutaneous adipose tissue. As a result, facial aging features become more pronounced, including increased skin laxity and the deepening of dynamic and static wrinkles. These changes are particularly evident in anatomically vulnerable areas such as the temples, cheeks, tear troughs, melomental folds, and nasolabial folds. Additionally, the zygomatic bones may appear more prominent, and the periorbital region often takes on a hollowed or sunken appearance.

These facial changes are likely not exclusive to semaglutide, but rather common to other agents within the same pharmacological class—namely glucagon-like peptide-1 receptor agonists (GLP-1 RAs)—including tirzepatide (commercially known as Mounjaro), among others. Moreover, similar facial alterations can occur following any form of significant and rapid weight loss, regardless of etiology. In both pharmacological and non-pharmacological contexts, the term *“Ozempic face”* has become a widely adopted colloquial descriptor for this constellation of aesthetic changes, even when semaglutide is not the direct causative agent.

We present a pilot study that reports our clinical experience in the treatment of *“Ozempic face”* using subdermal bipolar radiofrequency. Specifically, we utilized the BodyTite device, supplied by InMode Italy S.r.l., which allowed for the effective management of multiple clinical cases in a minimally invasive manner. This approach enabled timely aesthetic restoration while avoiding more aggressive surgical interventions and substantially reducing treatment-related costs for patients.

### 1.1. GLP-1 Receptor Agonists (GLP-1 RAs)

Glucagon-like peptide-1 receptor agonists (GLP-1 RAs) are a class of antidiabetic medications recently approved for the management of Type 2 Diabetes Mellitus. They are increasingly being adopted as a preferred alternative to traditional hypoglycemic agents, such as thiazolidinediones, sulfonylureas, and exogenous insulin. GLP-1 RAs are progressively replacing these conventional therapies due to their favorable safety profile, particularly the markedly lower risk of hypoglycemia and the additional benefit of promoting weight loss, rather than weight gain—an adverse effect commonly associated with older antidiabetic drugs [1].

GLP-1, or glucagon-like peptide-1, is an endogenous hormone secreted by intestinal L-cells in response to nutrient ingestion. It belongs to the incretin family—a group of gut-derived peptides that potentiate glucose-dependent insulin secretion. Among the incretins, the two most physiologically significant are GLP-1 and GIP (glucose-dependent insulinotropic polypeptide, formerly known as gastric inhibitory polypeptide). These hormones play a key role in postprandial glucose homeostasis by enhancing insulin release, suppressing glucagon secretion, and modulating gastric emptying.

*Incretins* exert a broad range of beneficial effects on human physiology. In particular, GLP-1 mediates its actions via the GLP-1 receptor (GLP-1R), which is expressed in a diverse array of tissues, including the brain, pancreas, cardiovascular system, liver, kidneys, stomach, skeletal muscle, adipose tissue, and likely additional sites that have yet to be fully characterized.

Among its most well-established physiological functions, GLP-1 plays a pivotal role in the regulation of glucose homeostasis. It enhances insulin secretion through direct stimulation of pancreatic β-cells, while simultaneously suppressing glucagon release by inhibiting pancreatic α-cells [2]. Additionally, GLP-1 contributes to weight loss through multiple mechanisms: it delays gastric emptying—thereby prolonging postprandial satiety—and modulates appetite by acting on hypothalamic centers involved in energy balance and hunger regulation [3].

A growing body of evidence has documented numerous additional physiological effects of GLP-1, particularly its cardiovascular and systemic benefits. GLP-1 receptor agonists (GLP-1 RAs) have been shown to reduce the incidence of major adverse cardiovascular events (MACEs) in patients with Type 2 Diabetes Mellitus [4]. These agents also exert beneficial effects on blood pressure regulation, contributing to the reduction of hypertension [5], and have demonstrated renoprotective properties, including the enhancement of renal function and the attenuation of albuminuria [6]. Furthermore, GLP-1 displays notable anti-inflammatory and immunomodulatory activities, which may further contribute to its systemic therapeutic potential beyond glycemic control [7].

GLP-1 receptors (GLP-1Rs) are G protein-coupled receptors (GPCRs) expressed on the plasma membranes of various cell types. Upon the binding of the GLP-1 ligand to the receptor’s extracellular domain, a complex intracellular signaling cascade is initiated, leading to cell type-specific physiological effects. In pancreatic β-cells, the activation of GLP-1Rs promotes insulin gene transcription and biosynthesis. However, insulin secretion remains tightly regulated by circulating glucose levels. This glucose-dependent mechanism of action represents a major therapeutic advantage of GLP-1 receptor agonists, as it substantially reduces the risk of hypoglycemia—a common adverse effect of many traditional antidiabetic agents [8].

Importantly, in cases where pancreatic β-cells have lost the capacity to produce insulin, activation of the GLP-1 receptor is rendered ineffective. Consequently, GLP-1 receptor agonists are approved exclusively for the treatment of Type 2 Diabetes Mellitus [9]. Furthermore, due to their weight-reducing properties, these agents have also gained regulatory approval for the management of obesity in individuals with obesity-related comorbidities [10].

GLP-1 receptor agonists can be classified into two categories based on their pharmacokinetic profiles:

- Long-acting GLP-1 Ras;
- Short-acting GLP-1 RAs.

Notably, exenatide is available in both short- and long-acting forms. Administration of these agents is primarily via subcutaneous injection, with the exception of semaglutide, which is uniquely available in both subcutaneous (Ozempic) and oral (Rybelsus) formulations.

Long-acting GLP-1 receptor agonists are typically administered once weekly and include the following agents:

- Exenatide (Bydureon),
- Dulaglutide (Trulicity),
- Semaglutide (Ozempic and Wegovy),
- Tirzepatide (Mounjaro).

Tirzepatide is often classified among GLP-1 receptor agonists; however, it exerts a dual pharmacological effect by concurrently targeting both the GLP-1 receptor (GLP-1R) and the glucose-dependent insulinotropic polypeptide receptor (GIPR) [11].

Short-acting GLP-1 RAs are generally administrated once daily, with the exception of exenatide (Byetta), which requires twice-daily administration.

This category includes the following:

- Exenatide (Byetta),
- Lixisenatide (Lyxumia),
- Liraglutide (Saxenda),
- Semaglutide (Rybelsus, oral formulation).

Multiple clinical studies have demonstrated that long-acting GLP-1 receptor agonists are associated with significantly greater weight loss compared to their short-acting counterparts. These findings highlight the sustained physiological benefits of prolonged receptor activation, as such benefits contribute to improved appetite regulation and enhanced long-term metabolic control [12,13,14].

*Saxenda* (liraglutide), *Wegovy* (semaglutide), and *Mounjaro* (tirzepatide) have received approval from both the U.S. Food and Drug Administration (FDA) and the European Medicines Agency (EMA) for the treatment of obesity in individuals with a body mass index (BMI) ≥ 30 kg/m^2^, or in those with a BMI between 27 and 30 kg/m^2^ who present with at least one weight-related comorbidity, such as Type 2 Diabetes Mellitus, hypertension, hyperlipidemia, or obstructive sleep apnea syndrome (OSAS) [15,16]. Tirzepatide is marketed in the United States under the brand name *Zepbound*, whereas in the European Union, it is sold as Mounjaro for both Type 2 Diabetes and obesity. According to EMA regulatory standards, a single brand name is maintained across indications when the dosing regimen remains consistent.

*Byetta and Byuderon* (exenatide) [17], *Victoza* (liraglutide) [18], *Trulicity* (dulaglutide) [19], *Lyxumia* (lixisenatide) [20], and *Ozempic and Rybelsus* (semaglutide) [21] have all received regulatory approval from both the U.S. FDA and EMA for the treatment of Type 2 Diabetes Mellitus.

Semaglutide has demonstrated significant clinical benefits, as evidenced by four recent randomized, placebo-controlled trials. These include the SELECT, STEP-HFpEF, STEP-HFpEF DM, and FLOW studies, each enrolling distinct patient populations: individuals with atherosclerotic cardiovascular disease and overweight or obesity (SELECT); patients with a history of heart failure with mildly reduced or preserved ejection fraction (STEP-HFpEF); patients with heart failure with preserved ejection fraction and Type 2 Diabetes Mellitus (STEP-HFpEF DM); and individuals with Type 2 Diabetes Mellitus and chronic kidney disease (FLOW). Collectively, these trials have shown that treatment with semaglutide, compared to placebo, significantly reduces the risk of cardiovascular death and worsening heart failure events in these patient cohorts [22].

Studies such as STEP-1 [23], STEP-2 [24], STEP-3 [25], and SURMOUNT-1 [26] have demonstrated weight reductions ranging from 10% to 20%, with a substantial proportion—up to 75–85%—of this loss attributable to reductions in fat mass. Concurrently, an average reduction of approximately 20% in lean muscle mass has also been observed.

Lean body mass, particularly muscle mass, decreases as well, albeit to a lesser extent than fat mass. The proportion of lean mass loss relative to total body weight reduction typically ranges between 20% and 25% [27].

Currently, five pharmacological agents have been approved by the European Medicines Agency (EMA) for the treatment of obesity: orlistat, semaglutide, liraglutide, naltrexone/bupropion, and tirzepatide [28]. Setmelanotide is another anti-obesity medication; however, it has been approved only for three ultra-rare genetic disorders that cause obesity: Pro-OpioMelanoCortin (POMC) deficiency, Proprotein Convertase Subtilisin and Kexin type 1 (PCSK1) deficiency, and LEPtin Receptor (LEPR) deficiency [29].

However, off-label prescribing of these agents is not uncommon. For instance, although only Wegovy (semaglutide) is officially approved for obesity management, Ozempic—approved for Type 2 Diabetes Mellitus—is frequently used off label for weight reduction. Moreover, Ozempic is often prescribed without concurrent recommendations for lifestyle modifications, such as increased physical activity and caloric restriction [30].

The prescription of these agents has markedly increased in both Europe and the United States over the past year, despite limited comprehensive data on their side-effect profiles within the general population. Data from EudraVigilance concerning severe adverse events associated with anti-obesity medications reveal that the annual number of reports related to semaglutide has continued to rise since its widespread adoption, whereas reports for other anti-obesity agents have either declined or ceased entirely. Conversely, data on non-serious adverse events show an increase solely for liraglutide, while reports for other agents, including semaglutide, have decreased [30].

### 1.2. Bipolar Radiofrequency (RF)

Bipolar radiofrequency (RF) represents a recent technological advancement that has been increasingly adopted across multiple medical specialties, including cardiology [31] and orthopedics [32].

Nonetheless, its most widespread application is within the field of aesthetic medicine, where bipolar RF is predominantly utilized for facial-contouring procedures [33,34,35].

The fundamental mechanism underlying the use of radiofrequency (RF) in facial contouring involves the stimulation of collagen synthesis and the remodeling of connective tissue through an **endogenous** thermal effect. The term “endogenous” indicates that RF does not generate heat directly; instead, heat is produced indirectly as a result of the oscillation of intracellular water molecules and electrolytes. This internally generated heat is non-damaging to biological tissues, provides greater precision compared to exogenous heat sources, and enhances cellular metabolism. Ultimately, these effects promote neocollagenesis and soft tissue remodeling [36].

Endotissutal Bipolar Radiofrequency (RF) represents an advancement of the traditional RF technique, involving the insertion of one electrode into the subcutaneous tissue while the second electrode remains external. Due to the extreme thinness of the internal electrode, Endotissutal Bipolar RF is minimally invasive yet can be considered a viable alternative to conventional surgical procedures [37].

The primary advantages of Endotissutal Bipolar Radiofrequency include the following:

- Minimal invasiveness,
- Low cost,
- Few complications,
- Repeatability,
- Absence of contraindications.

### 1.3. Physical Principles of Bipolar Radiofrequency (RF) and Clinical Applications

Radiofrequency (RF) waves are electromagnetic oscillations composed of alternating electric and magnetic fields that propagate through space. These waves do not require a physical medium for transmission and travel at the speed of light—approximately 300,000 km/s in a vacuum.

Electromagnetic waves are typically classified according to their frequency and wavelength. **Frequency** refers to the number of wave oscillations occurring per second and is measured in Hertz (Hz), while **wavelength** denotes the spatial distance between two successive points on a wave—such as two adjacent peaks—and is commonly represented by the Greek letter λ (lambda). Frequency and wavelength are inversely related, and their relationship is governed by the following equation:

λ = c/f

This implies that wavelength is defined as the ratio between the speed of light (c) and the wave’s frequency (f), expressed by the following equation: λ = c/f. Radio waves are characterized by frequencies equal to or below 250 MHz and wavelengths ranging from approximately 10 cm to 10 km. They occupy the lowest frequency range and, correspondingly, the longest wavelengths within the electromagnetic spectrum. Notably, radio waves are entirely safe for human exposure, as they are non-ionizing and therefore lack the energy required to alter cellular DNA.

Bipolar radiofrequency (RF) is a technology that generates radio waves by establishing a potential difference between two electrodes—one positively charged and the other negatively charged. The resulting electric current propagates in the form of a wave at a frequency of approximately one million cycles per second (1 MHz) between the two poles. When this energy is delivered through a conductive medium, such as human tissue, it induces the oscillation of water molecules and electrolytes, thereby generating heat through an endogenous mechanism.

**Figure 1:**
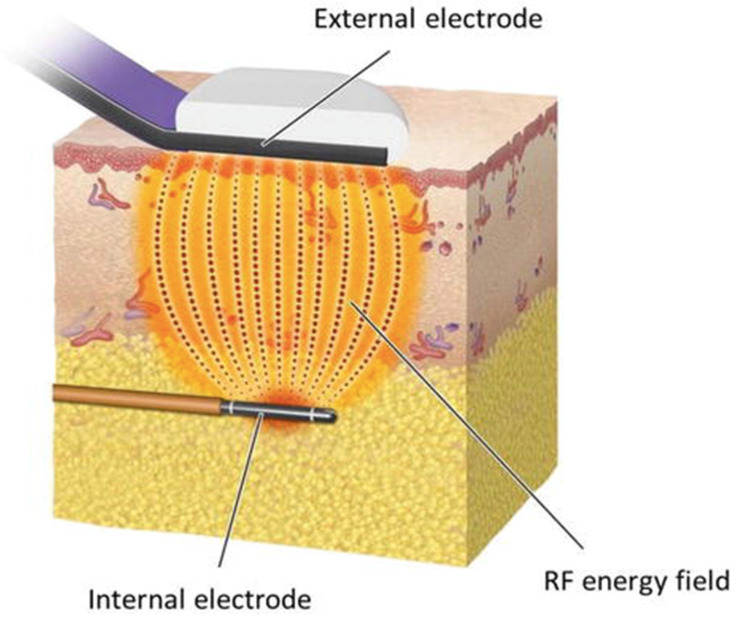
Graphic representation of Endotissutal Bipolar RF. The internal electrode generates heat that progressively decreases as it diffuses toward the surface, and the dermal layer is exposed to non-ablative thermal energy [20].

Endotissutal Bipolar Radiofrequency is a non-ablative aesthetic procedure designed to remodel the facial profile by selectively targeting and stimulating the most superficial layers of the dermis (Figure 1).

The procedure is facilitated by a handpiece consisting of two electrodes: one internal and one external. The internal electrode is positively charged, whereas the external electrode carries a negative charge. The potential difference between the two generates a bipolar RF current that oscillates between the internal (positive) and external (negative) electrodes. The intensity of the RF energy is greatest at its point of origin—within approximately 1–2 cm of the internal electrode—and progressively decreases as it propagates toward the external electrode. Consequently, the dermal layer, which lies closer to the external electrode, undergoes non-ablative thermal stimulation.

The handpiece is composed of an internal electrode in the form of a silicon-coated cannula, featuring an exposed tip that emits radiofrequency (RF) energy. The distal end of the internal electrode is bullet-shaped to facilitate dissection of soft tissues and to enable smooth navigation of the tip within the subcutaneous adipose layer. The external electrode, button-shaped in design, is located at the distal end of an arm positioned parallel to the internal electrode. It functions as the return pole, receiving the RF energy transmitted through the tissue from the internal electrode. By the time the RF waves reach the external electrode, the majority of their energy has already been dissipated in proximity to the internal electrode. As a result, the effect on the dermis is limited to inducing tissue remodeling, thereby counteracting skin laxity.

## 2. Materials and Methods

**Figure 2:**
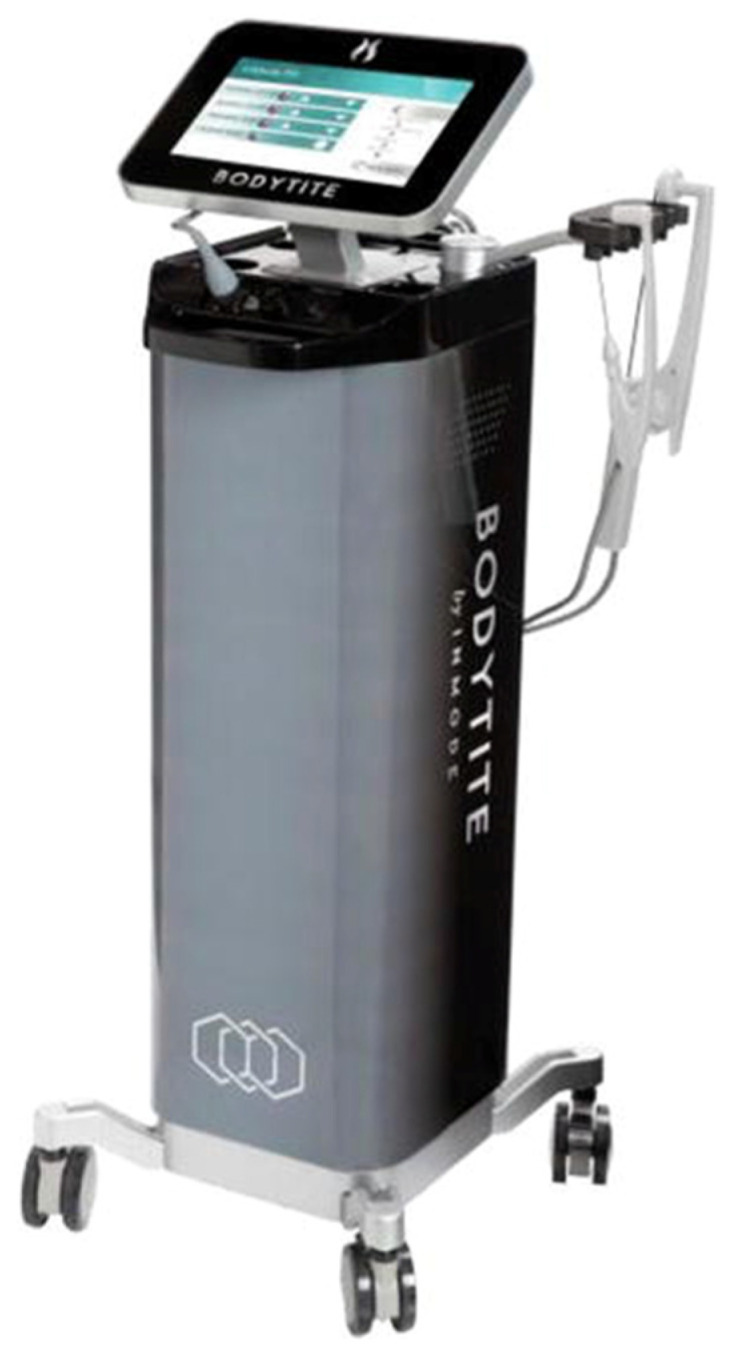
The BodyTite device, featuring its control panel and the handpiece composed of external and internal electrodes.

In this article, we present our two-year experience with RF-assisted facial contouring in patients exhibiting the so-called “Ozempic face,” using the BodyTite device supplied by InMode Italy S.r.l. (Rome, Italy) (Figure 2).

Over the past two years, we have treated 24 patients using this procedure, including 19 women and 5 men, ranging in age from 27 to 65 years (mean age: 43.6 years). Complications were infrequent and limited to postoperative erythema observed in four patients. In three cases, the erythema resolved spontaneously within five days, whereas in one patient, it persisted and required treatment with a topical corticosteroid ointment. In this latter case, complete resolution was achieved within an additional two days.

No other complications were observed. All patients wore a compression dressing for the first 48 h post-procedure, which effectively helped prevent the development of postoperative edema. Postoperative pain was well controlled with analgesics, predominantly paracetamol in all cases.

The outcomes of the procedure were evaluated 12 months post-treatment using two assessment criteria: patient self-assessment through a satisfaction score regarding the results at one year, and an independent expert evaluation of the stability of the aesthetic outcome over the same period. Specifically, patients were recalled 12 months after the intervention to complete a self-assessment questionnaire, at the end of which they rated their overall satisfaction on a scale from 0 to 10. Subsequently, each patient underwent an in-person examination by an independent expert who compared preoperative photographs with the current facial appearance at the 12-month follow-up. The expert assigned a score, also on a 0-to-10 scale, reflecting the perceived stability of the aesthetic result. The assessment focused on the persistence of treatment effects across four strategic facial regions:

1. Submandibular area,
2. Submental area,
3. Melolabial fold,
4. Labiomental sulcus.

Specifically, the expert evaluated the persistence of smoothing in the nasolabial and labiomental folds, as well as the maintenance of soft-tissue tone in the submandibular and submental regions of the neck. Each of these four parameters was scored on a scale from 0 to 10, and an average score was subsequently calculated.

## 3. Metabolic Analysis of the Sample

The term “Ozempic face” denotes the facial aging changes that may occur following rapid weight loss, regardless of whether the weight reduction arises from pharmacologic intervention or voluntary caloric restriction. Consequently, participant selection for this study was deliberately heterogeneous, encompassing individuals who experienced weight loss attributable to semaglutide (Ozempic), those who achieved weight reduction through self-directed dietary measures, and those whose weight loss resulted from alternative anti-obesity pharmacotherapies.

Table 1 summarizes the metabolic profiles of the patients enrolled in the study. The aim was to assemble a cohort with maximal heterogeneity. As illustrated, approximately half of the participants had a clinical diagnosis of Diabetes Mellitus, while the remainder were classified as obese in the absence of diabetes. Notably, all diabetic individuals were also overweight. Three obese, non-diabetic patients received GLP-1 receptor agonists off-label—that is, agents not (yet) formally approved for the treatment of obesity. Specifically, two patients were treated with semaglutide (Ozempic) and one with liraglutide (Victoza). In these cases, the medications were administered at the lowest available therapeutic dose to mitigate the risk of adverse effects.

Three obese participants in the study were treated with GLP-1 receptor agonists that are approved for the management of obesity. Of these, two received semaglutide (Wegovy), and one was treated with liraglutide (Saxenda). The remaining obese individuals were managed exclusively through a calorie-restricted dietary regimen.

The duration of treatment prior to undergoing radiofrequency therapy ranged from 8 to 50 weeks, with a mean duration of 28.5 weeks.

All individuals demonstrated weight loss relative to their baseline body weight, with reductions ranging from 5% to 14%, and a mean percentage decrease of 8.5%.

The absolute weight loss ranged from a minimum of 5.0 kg to a maximum of 20.7 kg, with a mean reduction of 9.8 kg.

Table 1 also includes data related to lifestyle modifications, specifically indicating whether patients incorporated regular physical activity into their routines—defined as either daily exercise or a minimum frequency of three sessions per week (thirty minutes of brisk walking per day).

As shown, the majority of patients combined pharmacological treatment or dietary intervention with regular physical activity, defined as low-to-moderate-intensity exercise performed either daily or at a minimum frequency of three times per week.

Only five subjects declined to modify their lifestyle by incorporating regular physical activity.

Among these, two individuals were managed exclusively through dietary intervention, without concomitant pharmacological treatment. Notably, in the absence of regular physical activity, the magnitude of weight loss observed was comparatively diminished.

All subjects receiving pharmacological therapy concurrently adhered to a calorie-restricted dietary regimen.

Bioelectrical impedance analysis was performed on eleven individuals at baseline and immediately prior to undergoing radiofrequency therapy.

In all cases, an improvement in the fat-mass-to-lean-mass ratio was observed.

In these patients, bioimpedance analysis was performed immediately prior to the initiation of anti-obesity therapy (either dietary or pharmacological), and was subsequently repeated at the end of the period indicated in the table (duration), corresponding to the time point immediately preceding treatment with radiofrequency therapy.

Table 2 reports the initial and final values of bioimpedance analysis. For each patient, the following information is presented: sex (first column); type of therapy administered (second column); duration of treatment (third column); weight loss in kilograms (fourth column); initial and final body weight (fifth column); the ratio of fat mass (FM) to muscle mass (MM, sixth column); initial and final fat mass (seventh column); initial and final Fat-Free Mass (FFM, eighth column); initial and final Visceral Adipose Tissue (VAT) (ninth column); initial and final muscle mass (tenth column); initial and final Skeletal Muscle Mass (SMM, eleventh column); initial and final Total Body Water (TBW, twelfth column); initial and final Extracellular Water (ECW, thirteenth column); initial and final Intracellular Water (ICW) (fourteenth column); and the initial and final Phase Angle (fifteenth column). Where both initial and final values are reported, the initial value is displayed above the final value.

## 4. Technical Procedure

**Figure 3:**
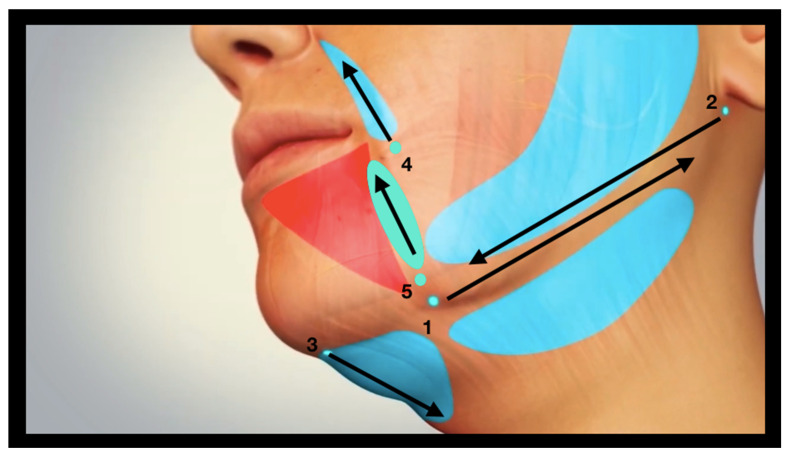
The four strategic regions targeted in the treatment of “Ozempic face” are the melolabial fold, the submandibular area, the submental area, and the labiomental sulcus.

**Figure 4:**
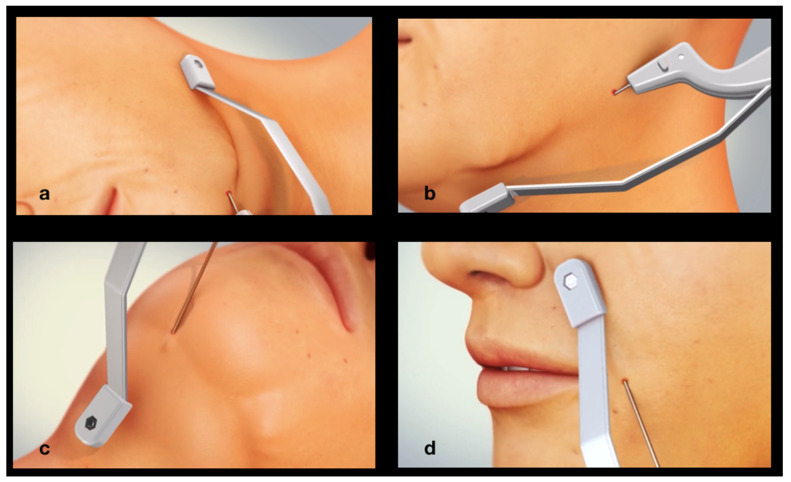
Treatment of the strategic zones: submandibular area (**a**,**b**), submental region (**c**), and nasolabial fold (**d**).

Figure 3 and Figure 4 depict the procedure of bipolar endo-tissular radiofrequency, a minimally invasive technique targeting four strategic facial regions for the treatment of “Ozempic face”:

1. Melolabial fold,
2. Labiomental sulcus,
3. Submandibular area,
4. Submental area.

Following administration of local anesthesia to the targeted skin area with 2% Mepivacaine containing Epinephrine (1:100,000), subcutaneous tissue access is achieved via a percutaneous entry point—without incision—using the metal tip of the lower electrode, which has a diameter of 21 Gauge.

For treatment of the submandibular region, the electrode tip is introduced via a dual-access approach at two distinct entry points. The first insertion is anterior, located where a vertical line drawn from the oral commissure intersects the inferior border of the mandible, corresponding anatomically to the insertion of the Depressor Labii Inferioris (DLI) muscle. From this point, the electrode is advanced posteriorly along the inferior mandibular border using a controlled to-and-fro motion. The second entry point is posterior, situated at the mandibular angle, from which the electrode is directed anteriorly toward the chin, again following the inferior mandibular margin with a to-and-fro movement.

Treatment of the submental region entails the insertion of the lower electrode tip at the midline of the chin. The electrode is subsequently advanced inferiorly through the subcutaneous soft tissues employing a controlled to-and-fro motion. A single entry point is deemed sufficient for effective coverage of the submental area.

Treatment of the melolabial fold involves needle insertion at its most caudal point (4). The electrode tip is subsequently advanced cranially along the trajectory of the fold using a controlled to-and-fro motion.

Finally, treatment of the labiomental sulcus involves needle insertion at its most caudal point (5). The electrode is then advanced cranially toward the oral commissure employing a controlled reciprocating motion.

In all cases, the electrode should be manipulated using both a to-and-fro motion and a lateral, fan-like sweeping movement, while maintaining its position within the subcutaneous plane, to ensure comprehensive coverage of the entire treatment area (highlighted in blue in Figure 3).

The internal electrode maintains a consistent temperature ranging from 60 to 70 °C, while the skin surface (external electrode) temperature is kept between approximately 38 and 40 °C. These temperature parameters are critical to the efficacy of radiofrequency treatment, as they remain below the threshold for tissue necrosis and instead promote tissue regeneration through controlled endogenous heating.

All patients received an identical treatment protocol, administered in three sessions spaced at 45-day intervals.

Figure 4a–d schematically illustrate needle insertion into the subcutaneous tissue at the strategic anatomical sites. The external electrode, positioned parallel to the internal electrode, is also clearly depicted. Both electrodes are rigidly connected via the handpiece and operate in a synchronized manner.

**Figure 5:**
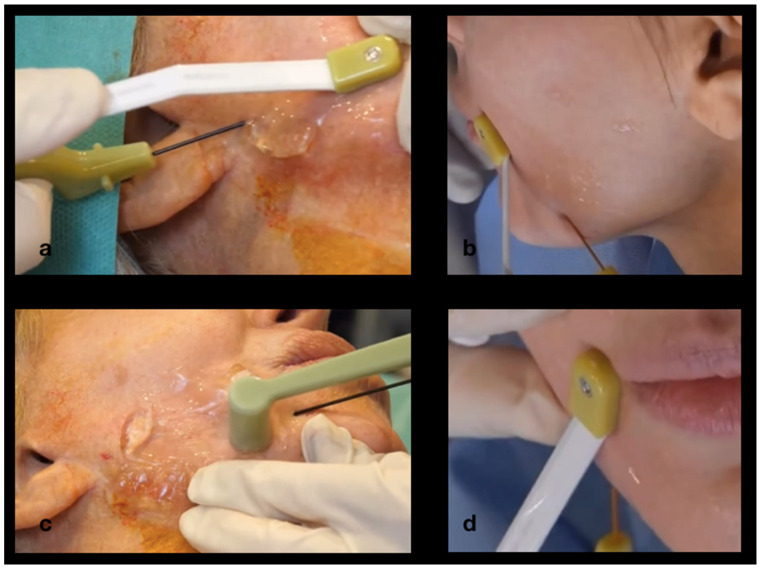
Intraoperative procedure (**a**–**d**).

Figure 5 presents an intraoperative image illustrating the procedure.

## 5. Results

As shown in Table 3, patients generally reported a high level of satisfaction with the aesthetic outcomes of the procedure. Three patients assigned the highest possible score (10/10); six reported near-maximal satisfaction (9/10); eight expressed satisfaction without reaching the highest level (8/10); five indicated moderate satisfaction, suggesting that slightly better results might have been attainable (7/10); and only two patients reported minimal satisfaction with the outcome (6/10).

**Figure 6:**
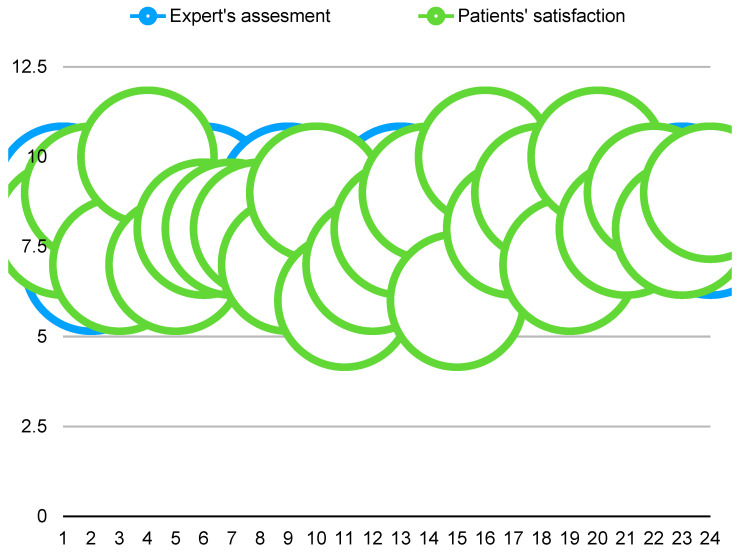
Green line: Patient satisfaction score (0–10); blue line: Expert’s evaluation of result stability 12 months post-treatment (0–10).

**Figure 7:**
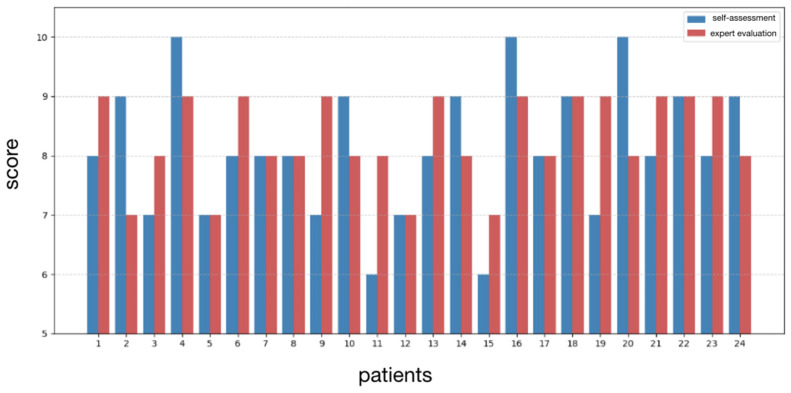
Histogram illustrating the frequency distribution of self-assessed aesthetic outcome scores by patients (blue), alongside expert’s evaluations of aesthetic result stability (red).

The expert’s evaluation of result stability closely correlates with patients’ subjective satisfaction, as illustrated in Figure 6 and Figure 7.

The results were subjected to statistical analysis for power estimation using a paired-sample *t*-test. The analysis yielded a mean difference (Δ) of 0.33, with a standard deviation (σ) of approximately 1.04, a Cohen’s d (effect size) of approximately −0.32, and a significance level (α) set at 0.05.

The statistical interpretation of these numerical findings enables us to draw the following considerations:

- A moderate degree of variability exists between patients’ self-reported satisfaction levels and the expert’s evaluations, as indicated by a standard deviation of approximately 1.04.
- Patients exhibited a tendency to slightly overestimate their outcomes compared to the expert’s assessment, as reflected by a Cohen’s d of approximately −0.32.

At a significance level (α) of 0.05, the calculated statistical power was 0.35, indicating that the sample size should be increased to achieve an adequately powered analysis. Nonetheless, this power level is considered acceptable for a pilot study such as the present investigation.

## 6. Clinical Cases

**Figure 8:**
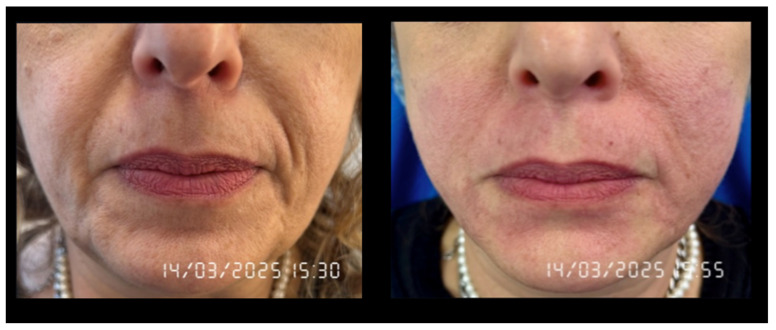
Patient’s appearance before (**left**) and after (**right**) Bipolar RF treatment; notable improvements are evident following a single session. The only side effect observed was localized erythema, which resolved spontaneously within five days.

An illustrative clinical case is presented below (Figure 8), involving a 54-year-old female patient. She had been receiving Ozempic since December 2024, following a diagnosis of Type 2 Diabetes Mellitus two months prior. Before initiating therapy, she was slightly overweight but generally satisfied with her physical appearance. However, after three months of treatment with Ozempic, despite experiencing weight loss, she reported noticeable alterations in her facial appearance and described herself as looking prematurely aged.

In Figure 8, the left image depicts the patient’s appearance prior to Bipolar RF treatment, whereas the right image clearly illustrates the significant improvement observed following a single session. The timing of both photographs underscores the immediate efficacy of the intervention: the skin appears more nourished and structurally enhanced, the depth of the labiomental lines and nasolabial folds is visibly reduced, and overall facial wrinkles are softened and substantially diminished. The sole complication noted was localized erythema in the treated area, which is clearly visible in the image and resolved spontaneously within five days.

The patient was advised to avoid direct sunlight exposure to the treated facial area and was prescribed a moisturizing cream for application as needed, whenever the skin appeared dry.

**Figure 9:**
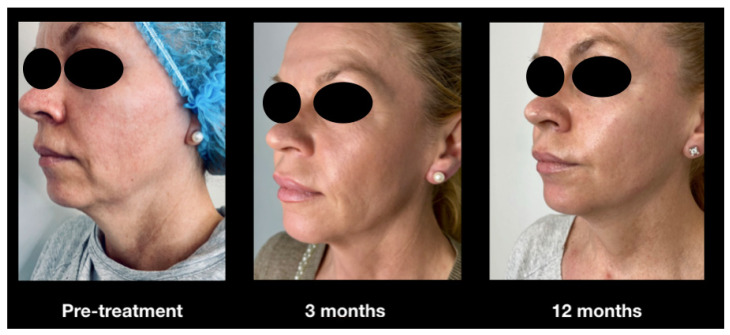
Clinical case of a female patient undergoing treatment with Ozempic for Type 2 Diabetes Mellitus. Notable findings included pronounced deepening of the nasolabial and labiomental folds, along with laxity of the cervical soft tissues. Significant aesthetic improvements were observed three months following radiofrequency (RF) treatment, with results well maintained at the 12-month follow-up.

Figure 9 illustrates a case of “Ozempic face” in a 52-year-old female patient undergoing treatment with semaglutide for Type 2 Diabetes Mellitus. The patient had been on the medication for eight months and presented herself to our clinic due to a markedly aged facial appearance compared to just a few months prior. Although she was not obese before initiating therapy, being only slightly overweight, signs of facial aging were already evident, including a pronounced nasolabial fold, a deep labiomental crease, and soft tissue laxity in the neck region.

Figure 9 also depicts the treatment effects at the three-month mark (central image), where noticeable smoothing of the nasolabial fold and labiomental crease is evident. Additionally, there is enhanced definition of the inferior mandibular margin and chin, attributable to reduced laxity of the cervical soft tissues. At the 12-month follow-up, these results appear to be well maintained.

**Figure 10:**
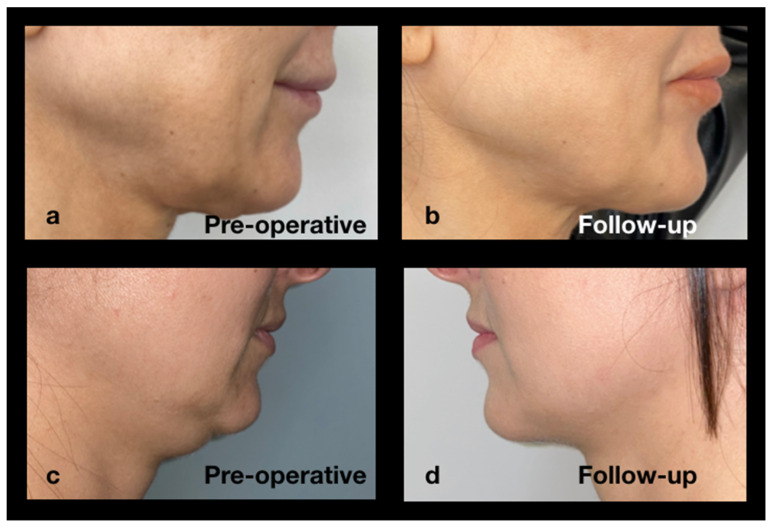
Preoperative profile and 12-month follow-up of two female patients (**a**–**d**) treated for “Ozempic face.”.

Figure 10 presents two additional clinical cases.

Top (a, b): A 42-year-old female patient undergoing treatment with Mounjaro for six months for Type 2 Diabetes Mellitus. Figure 10a shows the patient’s pre-treatment appearance, while Figure 10b depicts the 12-month follow-up post-treatment.

Bottom (c, d): A 37-year-old female patient undergoing treatment with Saxenda for four months for obesity. Figure 10c shows the pre-treatment profile, while Figure 10d depicts the appearance at the 12-month follow-up.

## 7. Discussion

Rapid weight loss, particularly when induced pharmacologically, often leads to a characteristic aging of the face, commonly referred to as “Ozempic face.” This condition is characterized by skin laxity, loss of facial volume, and deepening of facial folds, which occur due to the abrupt reduction of fat and changes in the collagen structure of the skin. While natural weight loss through diet and exercise can also affect facial appearance, the changes are typically more gradual and less pronounced. In contrast, pharmacological agents such as GLP-1 receptor agonists, including semaglutide (Ozempic), have become widespread treatments for obesity and Type 2 Diabetes. However, the rapid weight loss associated with these drugs can accelerate facial aging, creating aesthetic concerns for patients [38].

**Endotissutal Bipolar Radiofrequency (RF)** offers an advanced, minimally invasive solution to this issue. Unlike traditional radiofrequency techniques, which apply energy externally, Endotissutal Bipolar RF involves the insertion of one electrode into the subcutaneous tissue while the second electrode remains external. This approach directly targets the deeper dermal layers, inducing collagen remodeling and improving skin elasticity [39,40]. As such, it provides a promising alternative to more invasive procedures, such as rhytidectomy (facelift), for patients experiencing facial aging due to rapid weight loss. Furthermore, the technique’s minimal invasiveness, repeatability, and lower risk of complications make it an attractive option for non-surgical aesthetic enhancement [41].

Clinical studies have shown that bipolar RF, including **Endotissutal Bipolar RF**, is effective in treating skin laxity, improving skin texture, and reducing the appearance of wrinkles by stimulating collagen production [42,43]. These findings support the notion that **Endotissutal Bipolar RF** can counteract the detrimental aesthetic effects of rapid weight loss, including that associated with medications like semaglutide.

Additionally, **Endotissutal Bipolar RF** has been compared favorably to other non-invasive modalities, such as dermal fillers. While fillers provide temporary volume restoration, they do not address the underlying skin laxity, which can be a major concern for patients with “Ozempic face.” On the other hand, **Endotissutal Bipolar RF** promotes long-term skin rejuvenation by stimulating neocollagenesis, which enhances skin elasticity and tightens the tissue over time [44,45]. This effect has been shown to persist even after the conclusion of the treatment protocol, supporting the stability of the outcomes observed in our study.

**Endotissutal Bipolar RF** offers several advantages over traditional RF approaches, particularly in its ability to penetrate deeper tissues with minimal invasiveness. The technique targets the subcutaneous layer, where the most significant changes occur in patients experiencing facial aging from weight loss [46]. This approach also limits the risk of thermal damage to the skin surface while allowing for effective deep tissue remodeling. Several studies demonstrate that this type of controlled, endogenous heat production allows for more precise and safer treatments, reducing the risk of complications such as burns or excessive skin irritation [47]. Moreover, its repeatability and low complication rate make it an ideal choice for patients seeking consistent results with minimal recovery time.

In contrast, surgical options like facelifts or neck lifts carry inherent risks, including scarring, extended recovery periods, and potential complications. Non-invasive methods such as **Endotissutal Bipolar RF** present a viable alternative with a significantly lower risk profile. This makes it particularly advantageous for individuals seeking a minimally invasive option to improve their facial appearance following rapid weight loss due to pharmacological agents [48].

However, despite the promising results, there are limitations that must be considered. The relatively small sample size and short follow-up period in our study may limit the generalizability of the findings. Long-term studies with larger cohorts are needed to better understand the sustainability of the results and any potential delayed side effects. Furthermore, while the efficacy of **Endotissutal Bipolar RF** has been demonstrated in addressing skin laxity, more research is required to evaluate its effectiveness in more extensive facial aging or with other conditions that may contribute to aesthetic concerns.

In conclusion, **Endotissutal Bipolar RF** represents a promising treatment for individuals experiencing facial aging due to rapid weight loss, particularly in the context of pharmacological weight reduction treatments such as semaglutide. The technique offers significant improvements in skin firmness, elasticity, and overall appearance with minimal risk and downtime. Future studies with larger sample sizes and longer follow-up periods are essential to confirm the long-term benefits and optimal application of this innovative technology.

## 8. Limitations

The main limitation of our study is, undoubtedly, the small sample size, which reduces the ability to detect potential adverse effects. Moreover, the relatively short follow-up period (12 months) does not allow us to rule out the possible emergence of delayed side effects or the risk of long-term relapse.

These considerations justify why the present study is, and remains, a pilot study—one that will ideally be followed by a larger-scale investigation, potentially involving multiple specialized centers.

The lack of randomization in the sample also represents a significant limitation of the present study. Patients were selected based on the presence of more pronounced degrees of facial aging. However, the expert evaluation was conducted in a blinded manner: the evaluator was unaware of whether the patient had undergone pharmacological treatment, and if so, which drug had been administered. This approach was intended to ensure that the assessment remained as objective as possible.

## 9. Conclusions

In this article, we present our clinical experience in treating “Ozempic face” using Endotissutal Bipolar Radiofrequency (*BodyTite*, provided by *InMode Italy S.r.l.*).

Our experience with this device can be unequivocally regarded as positive. All patients reported a high level of satisfaction with the aesthetic outcomes, as was consistent with the assessments made by an experienced clinical evaluator.

The authors acknowledge the limited statistical power of the study, due to both the small sample size and the short follow-up period. However, studies involving larger patient cohorts and extended follow-up durations are planned for the near future.

## Figures and Tables

**Table 1:** Metabolic Profile of patients.

Patients	Cause of Weight Loss	Regular Physical Activity	Obesity/Diabetes	Duration	% of Weight Loss	Kg Lost	Bioimpedance Analysis *
**1**	Wegovy (1 mg/week)	No	Obesity	20 weeks	5%	6.4 kg	39/61% (initial)
							20/80% (end)
**2**	Ozempic (0.5 mg/week)	Yes	Diabetes	30 weeks	8%	9.2 kg	42/58 (initial)
							22/78% (end)
**3**	Diet	Yes	Obesity	50 weeks	12%	19 kg	43/57% (initial)
							23/77% (end)
**4**	Mounjaro (5 mg/week)	Yes	Diabetes	40 weeks	7%	8 kg	41/59 (initial)
							26/74 (end)
**5**	Diet	No	Obesity	15 weeks	5%	5.6 kg	N/A
**6**	Ozempic (0.25 mg/week)	Yes	Obesity (off label)	18 weeks	9%	11.6 kg	N/A
**7**	Diet	Yes	Obesity	34 weeks	14%	20.7 kg	40/60% (initial)
							19/81% (end)
**8**	Saxenda (3 mg/die)	No	Obesity	8 weeks	8%	6 kg	N/A
**9**	Diet	Yes	Obesity	10 weeks	8%	8 kg	41/59% (initial)
							23/77% (end)
**10**	Ozempic (1 mg/week)	Yes	Diabetes	30 weeks	6%	7 kg	N/A
**11**	Bydureon (2 mg/week)	Yes	Diabetes	40 weeks	10%	8 kg	N/A
**12**	Diet	Yes	Obesity	30 weeks	7%	7.8 kg	42/58% (initial)
							22/78% (end)
**13**	Ozempic (0.25 mg/week)	Yes	Obesity (off label)	20 weeks	10%	10 kg	N/A
**14**	Diet	No	Diabetes	28 weeks	5%	5 kg	38/62% (initial)
							32/68% (end)
**15**	Diet	Yes	Obesity	40 weeks	12%	9.4 kg	N/A
**16**	Wegovy (1 mg/week)	Yes	Obesity	32 weeks	14%	17.4 kg	40/60% (initial)
							28/72% (end)
**17**	Ozempic (1 mg/week)	Yes	Diabetes	40 weeks	7%	9.6 kg	N/A
**18**	Trulicity (1.5 mg/week)	No	Diabetes	20 weeks	5%	7 kg	N/A
**19**	Rybelsus (7 mg/day, per os)	Yes	Diabetes	35 weeks	12%	10 kg	N/A
**20**	Diet	Yes	Obesity	40 weeks	8%	7 kg	42/58% (initial)
							20/80% (end)
**21**	Ozempic (0.5 mg/week)	Yes	Diabetes	30 weeks	7%	9 kg	N/A
**22**	Mounjaro (5 mg/week)	Yes	Diabetes	25 weeks	10%	12 kg	N/A
**23**	Diet	Yes	Obesity	30 weeks	8%	10 kg	42/58% (initial)
							22/78% (end)
**24**	Victoza (0.6 mg/die)	Yes	Obesity (off label)	20 weeks	7%	12 kg	N/A

* Fat-to-lean-mass ratio; NA, not available.

**Table 2:** Initial and final bioimpedance parameters.

Patients (Sex)	Therapy	Duration	Kg Lost	Weight (kg)	FM/MM Ratio (%)	FM (kg)	FFM (kg)	VAT (kg)	MM (kg)	SMM (kg)	TBW (L)	ECW (L)	ICW (L)	Phase Angle
W	Wegovy (1 mg/week)	20 weeks	6.4	128	39–61	49.92	78.08	9.98	42.94	38.65	57.00	19.00	38.00	5.0°
				121.6	20–80	24.32	97.28	4.86	53.50	48.15	71.01	23.67	47.34	6.5°
W	Ozempic (0.5 mg/week)	30 weeks	9.2	115	42–58	48.3	66.7	7.25	56.7	36.9	48.7	16.1	32.6	4.6°
				105.8	22–78	23.28	92.52	3.49	70.1	45.6	60.2	19.9	40.3	5.8°
W	Mounjaro (5 mg/week)	40 weeks	8	114.3	41–59	46.86	67.4	7.03	57.3	37.2	49.2	16.2	33	4.7
				106.3	26–74	27.64	78.66	4.15	66.9	43.5	57.4	18.9	38.5	5.6°
M	Diet	10 weeks	8	100	41–59	41	59	8.2	53.1	37.2	43.1	14.2	28.9	5.4°
				92	23–77	21.16	70.84	4.23	63.8	44.7	51.7	17.1	34.6	6.4°
W	Diet	30 weeks	7.8	111.4	42–58	46.79	64.61	7.0	54.9	35.7	47.2	15.6	31.6	4.6°
				103.6	22–78	22.79	80.81	3.42	68.7	44.7	59.0	19.5	39.5	5.8°
M	Diet	50 weeks	19	158.3	43.57	67.94	90.06	13.6	81.1	56.8	65.7	21.7	44	5.0°
				139.3	23–77	31.97	107.03	6.39	96.3	67.4	78.1	25.8	52.3	6.5°
W	Diet	28 weeks	5	100	38–62	38	62	5.7	52.7	34.3	45.3	14.9	30.4	5.0°
				95	32–68	30.4	64.6	4.6	54.9	35.7	47.2	15.6	31.6	5.6°
W	Wegovy (1 mg/week)	32 weeks	17.4	124.3	40–60	49.72	74.58	7.46	63.4	41.2	54.4	17.9	36.5	4.8°
				106.9	28–72	29.93	76.97	4.49	65.4	42.5	56.2	18.5	37.7	5.9°
W	Diet	40 weeks	7	87.5	42–58	36.7	50.75	5.51	43.14	28.04	37.05	12.23	24.82	4.8°
				80.5	20–80	16.10	64.4	2.42	54.74	35.58	47.01	15.5	31.6	6°
W	Diet	30 weeks	10	125	42–58	52.5	72.5	8.34	57.3	28.3	53.2	20.33	32.9	4.7°
				115	22/78	25.3	89.7	4.2	71.3	37.2	65.6	9.3	26.3	5.8°
W	Diet	34 weeks	20.7	147.8	40–60	59.1	88.7	8.9	71.9	38.1	65.4	26.3	39.1	4.8°
				127.1	19–81	24.1	103.0	3.8	82.9	45.6	76.9	36.9	40.0	6.2°

**Table 3:** List of patients treated with Endotissutal Bipolar Radiofrequency; the fourth and fifth columns, respectively, present the patients’ satisfaction scores and the expert’s evaluation of the durability of treatment outcomes.

Patients	Gender	Age	Complications	Satisfaction	Stability
**1**	W	56	No	8/10	9/10
**2**	W	43	No	9/10	7/10
**3**	W	53	No	7/10	8/10
**4**	M	62	No	10/10	9/10
**5**	W	27	No	7/10	7/10
**6**	W	43	No	8/10	9/10
**7**	M	32	No	8/10	8/10
**8**	M	58	Erythema	8/10	8/10
**9**	W	45	No	7/10	9/10
**10**	W	60	No	9/10	8/10
**11**	W	57	Erythema	6/10	8/10
**12**	W	65	No	7/10	7/10
**13**	W	29	No	8/10	9/10
**14**	M	54	No	9/10	8/10
**15**	W	41	No	6/10	7/10
**16**	W	64	Erythema	10/10	9/10
**17**	W	58	No	8/10	8/10
**18**	W	62	No	9/10	9/10
**19**	M	33	No	7/10	9/10
**20**	W	47	No	10/10	8/10
**21**	W	52	Erythema	8/10	9/10
**22**	W	39	No	9/10	9/10
**23**	W	40	No	8/10	9/10
**24**	W	59	No	9/10	8/10

## Data Availability

The data presented in this study are available on request from the corresponding author.
